# Ornithine in chronic migraine therapy and allodynia pattern: comparison with dopaminergic activation

**DOI:** 10.1186/1129-2377-14-S1-P189

**Published:** 2013-02-21

**Authors:** M Nicolodi, M Nicolodi

**Affiliations:** 1 Foundation Prevention and Therapy Primary Pain, Italy

## 

Glutamate is converted to ornithine. Many enzyme reactions are involved in the inter-conversion of glutamate and ornithine by intestinal mucosa [1]. The modulation of the conversion might be a target in the final modulation of glutamatergic transmission. Since ornithine synthesis depends on glutamate, a larger amount of ornithine can explicate a feedback limiting effect on glutamatergic availability. A possible limited glutamatergic availability is here compared with a dopaminergic activation obtained by means of amantadine. In fact, both limited glutamatergic action and increased dopaminergic activity have been indicated to be crucial in analgesia determined at the level of anterior cingulate cortex (ACC) [2]. Experiences in chronic Migraine (M) therapy: in 107 chronic M sufferers (69 females, 38 males, mean age 33.8 + 4.1 SD) ornithine (500 mg twice a day for 3 months) induced an amelioration paralleling the amantadine induced-relief (100 mg amantadine/day for 3 months). Indeed, both the active compounds induced a decrease of the attacks of 40% p>0.001 versus 30-days wash-out and 30-days run-in periods A 14-days treatment induced a decrease (p>0.0001) of visceral/vascular hyperalgesia/allodynia rating -65% in a 0-10 VAS when comparing baseline values with the ones after both amantadine and ornithine administered in the aforementioned doses. These results suggest: a) that ornithine may act in the inteconversion of glutamate in all the tissues. Moreover, large doses of ornithine may induce a negative feedback in the mentioned interconversion. b) Dopaminergic and glutamatergic transmission, having opposite activity in pain processing in the ACC, that, in is turn, seems to have a crucial structure in pain dyshomeosthasis, seemingly act on the central mechanisms of chronic M.
